# The *in vitro* replication phenotype of hepatitis B virus (HBV) splice variants Sp3 and Sp9 and their impact on wild-type HBV replication

**DOI:** 10.1128/jvi.01538-23

**Published:** 2024-03-19

**Authors:** Laura C. McCoullough, Tomas Sadauskas, Vitina Sozzi, Kai Yan Mak, Hugh Mason, Margaret Littlejohn, Peter A. Revill

**Affiliations:** 1 Victorian Infectious Diseases Reference Laboratory, Royal Melbourne Hospital at the Peter Doherty Institute for Infection and Immunity, Melbourne, Australia; 2 Department of Microbiology and Immunology, University of Melbourne at the Peter Doherty Institute for Infection and Immunity, Melbourne, Australia; 3 Department of Infectious Diseases, University of Melbourne at the Peter Doherty Institute for Infection and Immunity, Melbourne, Australia; University of Southern California, Los Angeles, California, USA

**Keywords:** hepatitis B virus, hepatitis B splice variants, hepatitis B replication, hepatitis B novel fusion proteins

## Abstract

Prior to nuclear export, the hepatitis B virus (HBV) pregenomic RNA may be spliced by the host cell spliceosome to form shorter RNA sequences known as splice variants. Due to deletions in the open reading frames, splice variants may encode novel fusion proteins. Although not essential for HBV replication, the role of splice variants and their novel fusion proteins largely remains unknown. Some splice variants and their encoded novel fusion proteins have been shown to impair or promote wild-type HBV replication *in vitro,* and although splice variants Sp3 and Sp9 are two of the most common splice variants identified to date, their *in vitro* replication phenotype and their impact on wild-type HBV replication are unclear. Here, we utilize greater than genome-length Sp3 and Sp9 constructs to investigate their replication phenotype *in vitro*, and their impact on wild-type HBV replication. We show that Sp3 and Sp9 were incapable of autonomous replication, which was rescued by providing the polymerase and core proteins *in trans*. Furthermore, we showed that Sp3 had no impact on wild-type HBV replication, whereas Sp9 strongly reduced wild-type HBV replication in co-transfection experiments. Knocking out Sp9 novel precore-surface and core-surface fusion protein partially restored replication, suggesting that these proteins contributed to suppression of wild-type HBV replication, providing further insights into factors regulating HBV replication *in vitro*.

**IMPORTANCE:**

The role of hepatitis B virus (HBV) splice variants in HBV replication and pathogenesis currently remains largely unknown. However, HBV splice variants have been associated with the development of hepatocellular carcinoma, suggesting a role in HBV pathogenesis. Several *in vitro* co-transfection studies have shown that different splice variants have varying impacts on wild-type HBV replication, perhaps contributing to viral persistence. Furthermore, all splice variants are predicted to produce novel fusion proteins. Sp1 hepatitis B splice protein contributes to liver disease progression and apoptosis; however, the function of other HBV splice variant novel fusion proteins remains largely unknown. We show that Sp9 markedly impairs HBV replication in a cell culture co-transfection model, mediated by expression of Sp9 novel fusion proteins. In contrast, Sp3 had no effect on wild-type HBV replication. Together, these studies provide further insights into viral factors contributing to regulation of HBV replication.

## INTRODUCTION

Hepatitis B virus (HBV) is a pararetrovirus encoding a small 3.2kb partially double-stranded relaxed circular DNA (rcDNA) genome. The HBV genome contains four open reading frames (ORF): precore (PC)/core ORF, surface (S) ORF, X ORF, and polymerase (pol) ORF. Following infection of hepatocytes, rcDNA is transported to the nucleus where it is converted to a covalently closed circular DNA (cccDNA) minichromosome that forms the major replication template (1). cccDNA is transcribed into a greater than genome-length pregenomic RNA (pgRNA) intermediate and viral mRNAs. The pgRNA is exported from the nucleus in an unspliced form, where it is packaged into core particles, reverse transcribed by the viral pol, enveloped and exported from the cell. The pgRNA may also be spliced by the host cell spliceosome to form shorter RNA sequences which retain the encapsidation signal, and may be packaged into core particles, reverse transcribed by the viral pol, and exported from the cell as defective viral particles (2). Splice-derived RNA forms up to 30% of total cellular HBV RNAs and up to 3% of serum-derived RNA (3, 4), yet their role in HBV replication and/or pathogenesis remains largely unknown.

**Fig 1:**
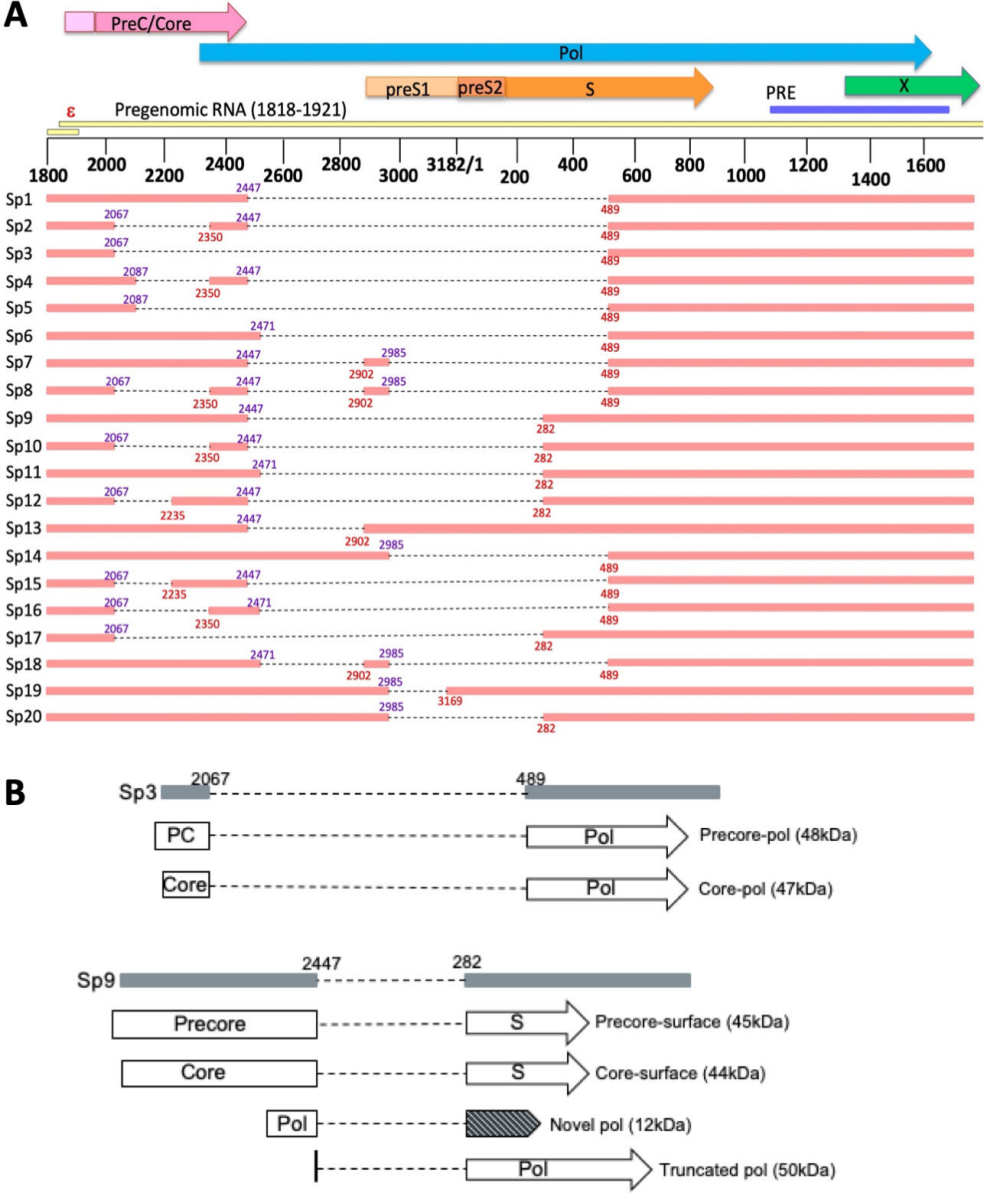
Characterized HBV splice variants and novel Sp3 and Sp9 fusion proteins. (**A) **Map of characterized HBV splice variants. Donor splice sites are indicated in purple and acceptor splice sites are indicated in red. Dotted lines indicate parts of genome missing due to splicing. Numbering is based on the EcoR1 start site. PreC/Core, precore/core open reading frame; pol, polymerase open reading frame; ε, epsilon encapsidation signal; PRE, post-regulatory element. (**B)** Map of predicted novel fusion proteins produced by Sp3 and Sp9. PC, precore. Shading indicates a new open reading frame.

Over 20 splice variants have been characterized and all identified splice variants have deletions in the pol open reading frame (Fig. 1A) (5–10). Therefore, they are incapable of autonomous replication; however, their replication may be rescued by wild-type (WT) pol protein provided *in trans* (3). Deletions in the open reading frames caused by removal of the splice intron mean that all splice variants are predicted to produce novel fusion proteins; however, only a small number of these have been characterized (11). The most commonly reported splice variant, Sp1, produces a novel pol protein termed the hepatitis B spliced protein, which is involved in cellular apoptosis and liver disease progression (12, 13). Sp1 also produces novel core, PC and C-terminal truncated pol proteins (14–16). Sp7 and Sp13 produce novel pol-S fusion proteins, and Sp10 produces a novel core-S fusion protein; however, the exact role of these proteins is unknown (17–19). All splice variants retain the full-length X ORF, and therefore encode the wild-type HBx protein.

While they are not required for HBV replication, the impact of splice variants on viral replication remains unclear. An increased proportion of splice variants in patient sera has been associated with liver disease and the development of hepatocellular carcinoma, perhaps suggesting a role in HBV pathogenesis (5, 20, 21). Furthermore, different splice variants have varying impacts on HBV replication in *in vitro* co-transfection experiments. Sp1, Sp10, and Sp13 decreased wild-type HBV replication, whereas Sp7 increased replication (15, 18, 22–24). Initial studies showed that the impact of Sp1 on wild-type HBV replication was mediated largely through the production of the novel PC protein, known as p21.5, via interactions with the nucleocapsid to impair capsid formation (15). However, our laboratory has recently shown that knocking out the Sp1 novel PC protein did not completely restore replication of wild-type HBV, suggesting other mechanisms may be involved (22). In contrast, impairment of wild-type HBV replication by Sp13 is largely mediated by the novel pol-S fusion protein, as knocking out this protein restored replication (24). Although not confirmed, Sp7 novel pol-S fusion protein may contribute to Sp7’s enhanced effect on replication, due to its transactivation activity (17). In contrast, Sp10 decreased wild-type HBV replication independently of the novel core-S fusion protein by acting as a non-coding RNA that interacted with the TATA-binding protein, preventing transcription (18). However, a limitation of these studies was that the splice constructs were driven by cytomegalovirus (CMV) promoters to overexpress the splice variant and did not reflect endogenous expression of splice RNA using natural HBV promoters. Furthermore, some studies used splice constructs that did not encode the full-length splice-derived sequence (15, 18). Ideally, constructs producing full-length splice RNA, driven by natural HBV promoters, should be used to investigate their replication phenotype and effect on HBV replication to better reflect their role in the HBV replication cycle. Our laboratory has recently used a full-length Sp1 construct to investigate its replication phenotype and effect on HBV replication *in vitro* (22).

Sp3, spliced between nucleotides (nt) 2067 and 489, and Sp9, spliced between nucleotides 2447 and 282, have both been reported as the second most common splice variant relative to Sp1 in different studies (3, 10, 25–29). Sp3 is predicted to produce a novel PC-pol and core-pol fusion protein, whereas Sp9 is predicted to produce a novel PC-S, core-S, novel pol, and an N-terminally truncated pol protein (Fig. 1B) (11). The replication phenotype of Sp3 and Sp9 and the impact of these splice variants and their novel fusion proteins on wild-type HBV replication are unknown. Here, we used full-length Sp3 and Sp9 constructs, which are driven by natural HBV promoters, to analyze the impact of Sp3 and Sp9 on wild-type HBV replication *in vitro*.

## MATERIALS AND METHODS

### 1.3mer WT HBV and splice variant DNA clones

1.3mer WT HBV genotype D3, WT HBV genotype A2, and HBV splice variant Sp1 clones used in this study have been previously described (22, 30). 1.3mer HBV splice variants clones Sp3 and Sp9 were derived from the WT HBV 1.3mer genotype D3 clone, by deleting the sequences of the introns corresponding to Sp3 (between nt 2067 and 489) and Sp9 (between nt 2447 and 282). 1.3mer HBV splice variant clone Sp10 was derived from the WT 1.3mer genotype A2 clone by deleting the sequences of the intron corresponding to Sp10 between nucleotides 2067 and 2350 and 2447 and 282. The sequences were synthesized by Genscript USA, ligated to pUC57 vector, and are expressed under the control of natural HBV promoters. A detailed map of each clone is shown in Fig. S1.

### Sp9 novel fusion protein expression clones and 1.3mer Sp9 mutant novel fusion protein knockout clones

#### Sp9 novel fusion protein sequences

Sp9 PC-S, core-S, PC-S without core-S, novel pol, and truncated pol were generated encoding a 3´ double FLAG tag in the CMV-driven pCI mammalian vector (Stratagene; Genscript USA).

#### 1.3mer Sp9 mutant clones

Sp9 novel fusion protein knockout clones were derived from the 1.3mer Sp9 clone by mutating nucleotides in the start codon of each fusion protein. Sp9 PC-S (A2766T), core-S (T2854A), PC-S and core-S (A2766T, T2854A), and novel pol fusion protein knockout clones (T3260C) were synthesized. A 1.3mer Sp9 clone that knocked out all the novel fusion proteins [A2766T (PC-S), T2854A (core-S), T3260C (novel pol), G3400T (truncated pol)] was also produced. HBV sequences were synthesized in a pUC57 vector (Genscript USA) and utilized endogenous HBV promoters. A detailed map of each clone is shown in Fig. S1.

### Rescue of splice variant replication

Replication of Sp1, Sp3, Sp9, and Sp10 was “rescued” by providing the HBV pol *in trans* as previously described using the replication competent HBV clone pCH3142 or an in-house CMV-driven Pol expression plasmid (22). pCH3142 (kindly provided by Prof. Hans Netter), which encodes a mutation in the epsilon encapsidation signal, is incapable of pgRNA packaging, but expresses the HBV pgRNA, mRNAs, and HBV proteins (31). The CMV-driven Pol expression plasmid was generated by inserting the HBV pol sequence from the WT 1.3mer D3 clone (Genscript, USA) into the CMV-driven pCI mammalian expression vector (Stratagene). The overlapping nature of the genome meant this plasmid also expressed the HBV S protein, but not the core or PC proteins.

### Cell culture and transfection

Huh7 or HepG2 cells were seeded to semi-confluence in 60 mm dishes, or 6- or 24-well plates, and allowed to adhere overnight. HepAD38 cells were seeded to semi-confluence in six-well plates. Transient transfections in Huh7 and HepG2 cells were performed using FuGENE 6 transfection reagent (Promega) according to the manufacturer’s instructions as previously described (32). Transient transfection in HepAD38 cells was performed using Lipofectamine 3000 (Thermofisher Scientific) according to the manufacturer’s instructions. Transfection efficiency was monitored using a green fluorescent protein (GFP) expression construct and counting GFP-expressing cells using a fluorescent microscope. Equal amounts of splice and rescue plasmid or WT HBV 1.3mer DNA were co-transfected to rescue replication of the splice constructs or determine their impact on HBV replication, respectively. pUC57 empty vector plasmid was included as filler DNA.

Intracellular core-associated HBV DNA was analyzed by Southern blotting from experiments performed using HepG2 cells, whereas intracellular HBV RNA and protein were analyzed by northern and western blotting from experiments performed using Huh7 cells, due to higher levels of RNA and protein expression from Huh7 transfections (32). All analyses were performed 5 days post-transfection when HBV DNA and protein expression was highest, except analysis of HBx and Sp9 novel fusion protein expression, which was performed 2 days post-transfection (32, 33).

### Analysis of intracellular nucleocapsid-associated HBV DNA

Cell harvest and extraction of HBV DNA were performed as previously described (34). Briefly, cell monolayers were lysed with a phosphate buffered saline containing 0.5% Nonidet P-40, transferred to microfuge tubes, and centrifuged to remove the nuclei pellet. Supernatants were then transferred to clean tubes. Contaminating input plasmid was digested with DNase I (Roche) and this step was repeated to ensure complete digestion of input plasmid. A solution containing 2.5% (wt/vol) SDS, 100 mM Tris pH 7.5, and 125 mM ethylenediaminetetraacetic acid (EDTA) and proteinase K (Roche) to a final concentration of 0.5 mg/mL were then added and allowed to incubate at 37°C overnight. DNA was extracted by sequential phenol-chloroform treatment and precipitated with isopropanol. DNA pellets were dissolved in nuclease-free water. HBV replicative intermediates were detected by electrophoresis and Southern blotting as previously described (34). Southern blots were probed with a digoxigenin (DIG)-labeled (Roche) 2.5 kb DNA probe, generated by PCR using forward and reverse primers (forward 5´AAGGTGGGAAACTTTACTGGGC3´; reverse 5´GGCAAAAACGAGAGTAACTC3´), using the 1.3mer D3 clone as template. This amplicon commenced downstream of the HBV core gene, and encompassed almost all of HBV Pol as well as the complete envelope and HBx genes.

### Total RNA

Total RNA was isolated from cell lysates using the RNeasy method (Qiagen) and HBV RNAs analyzed by northern hybridization as previously described (32, 34).

### Western blot analysis

Intracellular HBV proteins in cell lysates harvested 2 or 5 days post-transfection were detected by immunoblotting as previously described (35) using antibodies against the HBx (X36C, Santa Cruz sc-57760), HBsAg (H166 antibody kindly donated by Paul Coleman, Abbott Laboratories, Abbott Park, IL), and precore/core proteins (in-house monoclonal antibody 1D8) (36–39). Expression of Sp9 novel fusion proteins by the novel fusion protein expression plasmids was confirmed using an anti-flag antibody (Santa Cruz).

### Quantitative serology

HBeAg and HBsAg concentrations in cell culture lysates and supernatants were measured by chemiluminescent microparticle immunoassay (CMIA) using the Roche HBeAg and HBsAg assays on a Cobas e411 instrument as previously described (32).

### Cytotoxicity assay

The viability of transfected cells was determined using the CytoTox-Glo Cytotoxicity assay (Promega) as per the manufacturer’s instructions.

### Data analysis

Data analyses and graphs were performed in GraphPad Prism software version 7. Unpaired two-tailed Student’s *t*-test were performed. The *P*-values are indicated in the figures. The alpha value, *P* < 0.05, was considered statistically significant.

## RESULTS

### Sp3 and Sp9 replication was rescued by HBV pol and core supplied *in trans*

**Fig 2:**
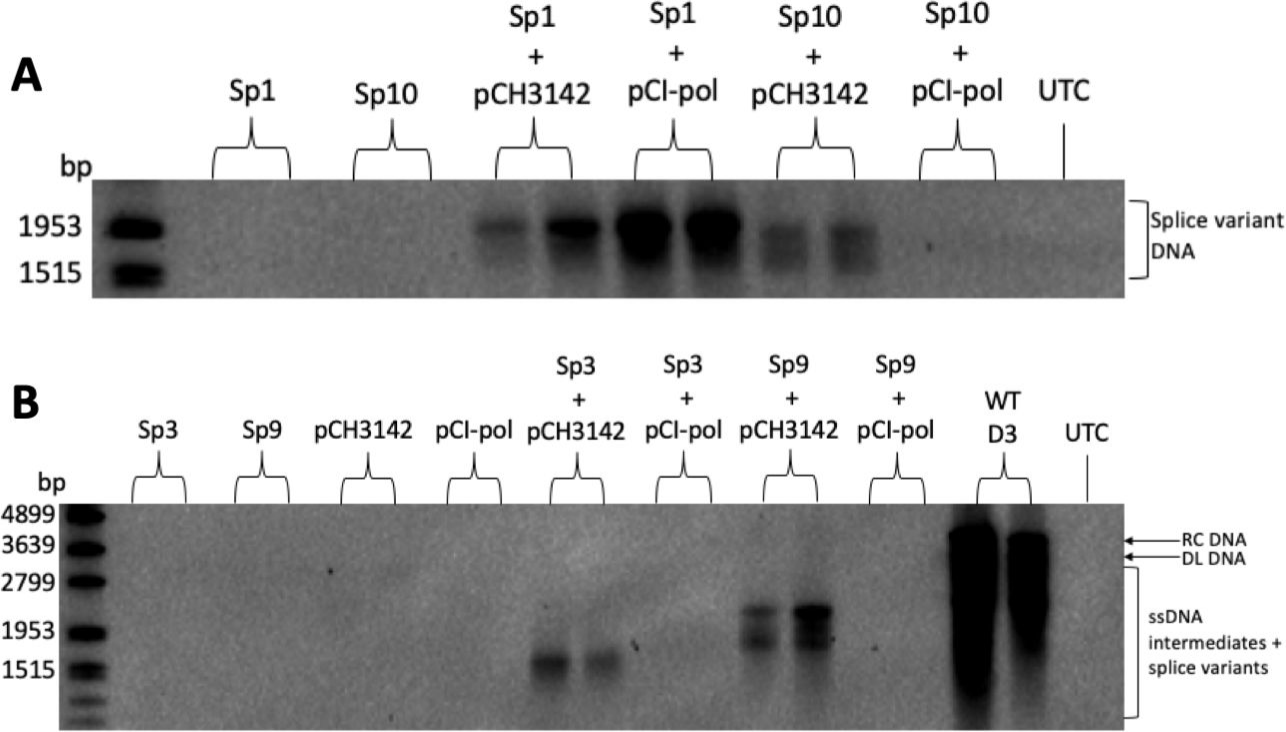
HBV splice variant replication was rescued by HBV proteins provided *in trans*. Southern blot of intracellular core-associated DNA 5 days post-transfection of plasmids expressing (**A**) Sp1 or Sp10 or (**B**) Sp3 or Sp9 with pCH3142 or pCI-pol alone. RC DNA, relaxed circular DNA; DL DNA, double-linear DNA; ssDNA, single-stranded DNA; UTC, untransfected control.

To confirm that HBV splice variants only replicate in the presence of HBV proteins provided *in trans*, Sp1, Sp3, Sp9, and Sp10 were transfected alone or with HBV protein-expressing pCH3142 or pCI-pol clones. As previously reported, Sp1 was incapable of autonomous replication (22) (Fig. 2A). However, replication was rescued by supplying the wild-type pol *in trans* by co-transfection of plasmids encoding the pol protein. Sp3, Sp9, and Sp10 were also incapable of autonomous replication (Fig. 2A and B). However, unlike Sp1, these splice variants also required the core protein to be supplied as rescue experiments using a plasmid which only expressed the pol and S proteins failed to support their replication, which required co-transfection of a plasmid expressing both the core and pol proteins.

### Sp9 expression inhibited wild-type HBV replication, whereas Sp3 had no impact

#### HBV DNA

**Fig 3:**
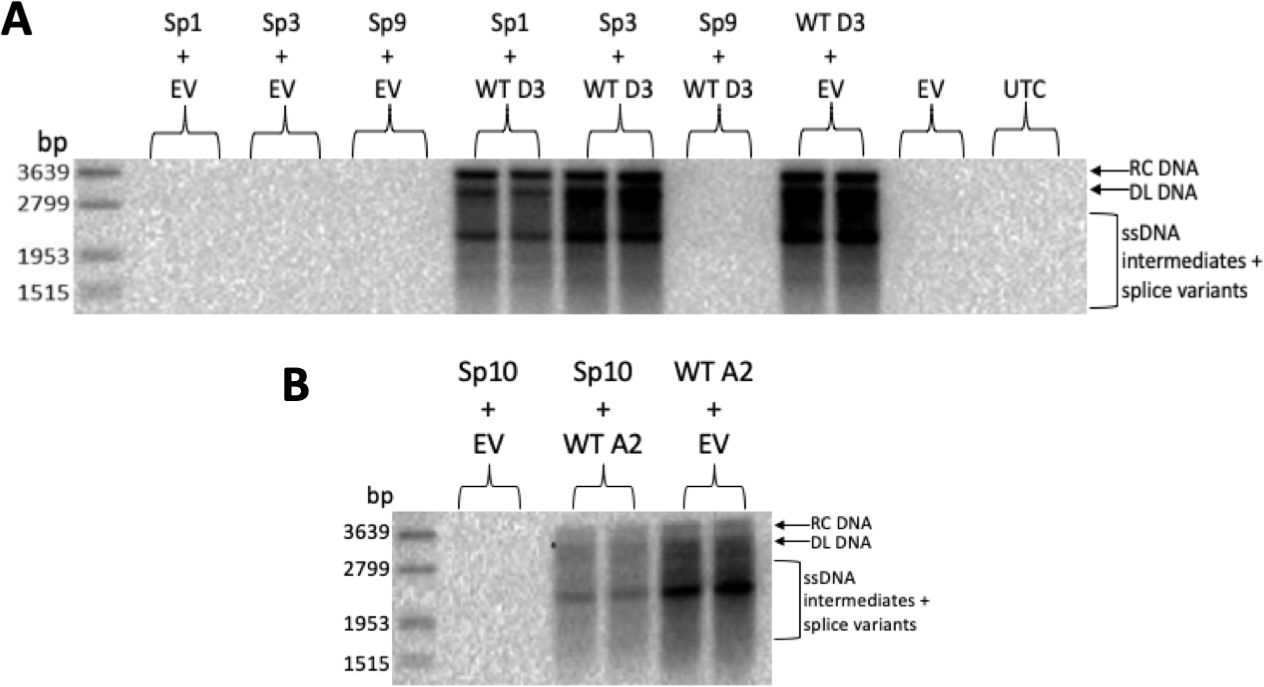
Sp9 strongly reduced intracellular core-associated HBV DNA. Southern blot of intracellular core-associated DNA 5 days post-transfection of plasmids expressing (**A**) Sp1, Sp3, Sp9, or (**B**) Sp10 with WT HBV. RC DNA, relaxed circular DNA; DL DNA, double-stranded linear DNA; ssDNA, single-stranded DNA; UTC, untransfected control; EV, empty vector.

To investigate the impact of Sp3 and Sp9 on wild-type HBV replication, Sp3 and Sp9 clones were co-transfected with WT HBV D3 clone. Sp1 and Sp10 were included as controls. As previously reported, co-transfection of Sp1 (Fig. 3A) or Sp10 (Fig. 3B) with WT DNA in equal concentration reduced intracellular core-associated wild-type HBV DNA, compared to co-transfection of WT DNA with an empty vector (18, 22). Co-transfection of Sp3 with WT DNA had no effect on intracellular core-associated wild-type HBV DNA. In contrast, co-transfection of Sp9 with WT DNA in equal concentration completely abrogated intracellular core-associated wild-type HBV DNA, compared to co-transfection of WT DNA with empty vector (Fig. 3A).

#### HBV RNA

**Fig 4:**
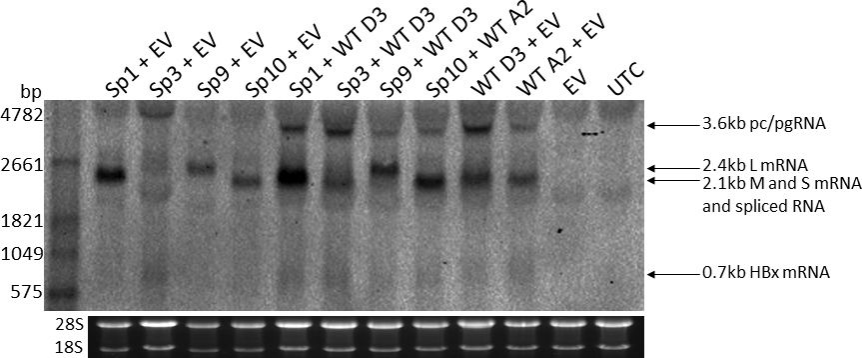
Sp9 reduced wild-type HBV intracellular RNA. Northern blot of intracellular RNA 5 days post-transfection of plasmids expressing Sp1, Sp3, Sp9, or Sp10 with WT HBV. pc/pgRNA, precore/pregenomic RNA; L, large surface; M, middle surface; S, small surface; HBx, X mRNA; EV, empty vector; UTC, untransfected control.

Analysis of intracellular RNA showed that, as previously reported, co-transfection of Sp1 and Sp10 with WT HBV reduced PC/pgRNA (18, 22) (Fig. 4). Sp3 had no impact on intracellular HBV RNA. In contrast, Sp9 co-transfected in equal proportions with WT HBV strongly reduced PC/pgRNA and S mRNA levels.

#### Intracellular and secreted protein expression

##### HBV core protein

**Fig 5:**
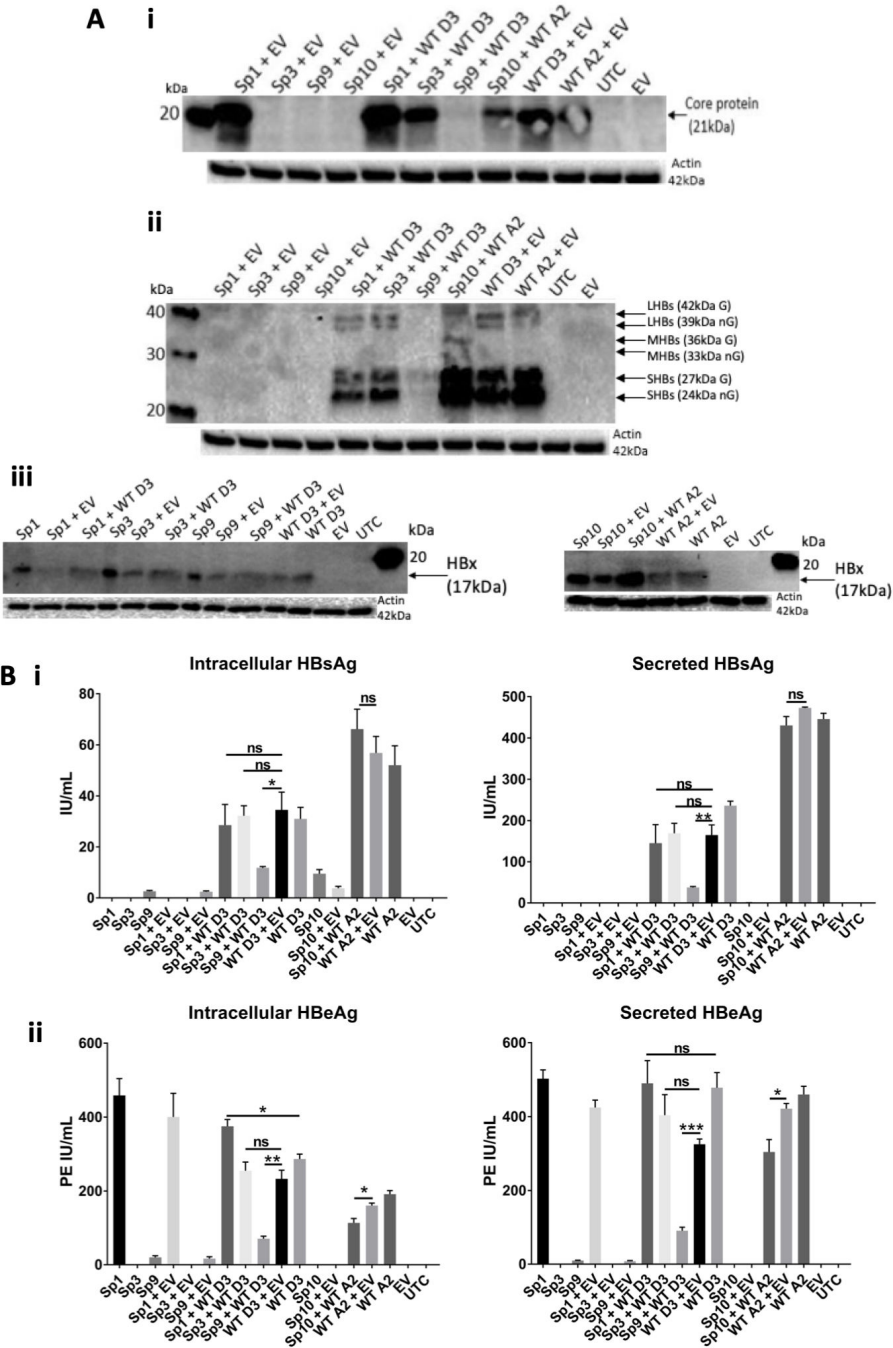
Sp9 strongly reduced wild-type intracellular and secreted HBV proteins. (**A)** Western blot of intracellular (i) core, probed with in-house monoclonal antibody 1D8 and (ii) HBsAg, probed with H166 antibody 5 days post-transfection and (iii) HBx, probed with X36C 2 days post-transfection of plasmids expressing Sp1, Sp3, Sp9, and Sp10 with WT HBV. LHBs, large HBV surface protein; MHBs, middle HBV surface protein; SHBs, small HBV surface protein. Note that the actin blot for (i) and (ii) are duplicates as they were from the same experiment. (**B)** Quantitative serology of intracellular and secreted (i) HBsAg and (ii) HBeAg expression 5 days post-transfection of plasmids expressing Sp1, Sp3,Sp9, and Sp10 with WT HBV. Error bars show SEM and *P*-values are produced from *t*-tests from three individual experiments. *, *P* < 0.05; **, *P* < 0.01; ***, *P* < 0.001; ns, not significant; PE, Paul-Ehrlich units; UTC, untransfected control; EV, empty vector.

Analysis of intracellular core protein expression showed that Sp1 was the only splice variant investigated that produced a core protein (Fig. 5A). Co-transfection of Sp1 with WT HBV increased core production, compared to WT transfected with empty vector, due to expression of Sp1 core protein. Co-transfection of Sp10 with WT HBV decreased core production. In contrast, Sp3 had no impact on core production, replicating the diluting effect of empty vector on wild-type HBV core protein levels. However, co-transfection of Sp9 with WT HBV strongly decreased core production, compared to WT/empty vector.

##### HBV S protein

As expected, all splice variants were incapable of producing S protein (Fig. 5). Co-transfection of Sp1, Sp3, or Sp10 with WT HBV had no significant effect on HBsAg production, as determined by quantitative serology (Fig. 5B). In contrast, Sp9 significantly decreased both intracellular and secreted HBsAg compared to WT with an empty vector (Fig. 5).

We failed to detect Sp9’s novel PC-S and core-S fusion proteins via western blotting; however, quantitative serology detected very low levels of intracellular S in cells transfected with Sp9 alone, which may be indicative of the production of its novel PC-S and/or core-S fusion proteins (Fig. 5B).

##### HBx protein

As expected, all splice variants produced HBx protein (Fig. 5A). No reduction in HBx expression was observed following co-transfection of the splice variants with WT HBV.

##### HBeAg expression

Intracellular and secreted HBeAg levels varied by splice variant, with highest levels detected for Sp1 and lowest for Sp3 and Sp10, which were incapable of expressing HBeAg (Fig. 5B). In a surprising finding, Sp9 expressed low levels of secreted HBeAg, likely expressed from the novel PC-S ORF (Fig. 5B). Co-transfection of Sp1 with WT HBV significantly increased intracellular HBeAg expression. In contrast, Sp3 had no effect on total HBeAg expression. Co-transfection of Sp9 or Sp10 with WT HBV significantly decreased total HBeAg expression, compared to WT with an empty vector (Fig. 5B).

### Cell viability assays

No significant difference was observed in cell viability between cells co-transfected with Sp1, Sp3, Sp9, or Sp10 with WT HBV compared to the untransfected control (Fig. S2).

### The impact of Sp9 on wild-type HBV replication was dose dependent

**Fig 6:**
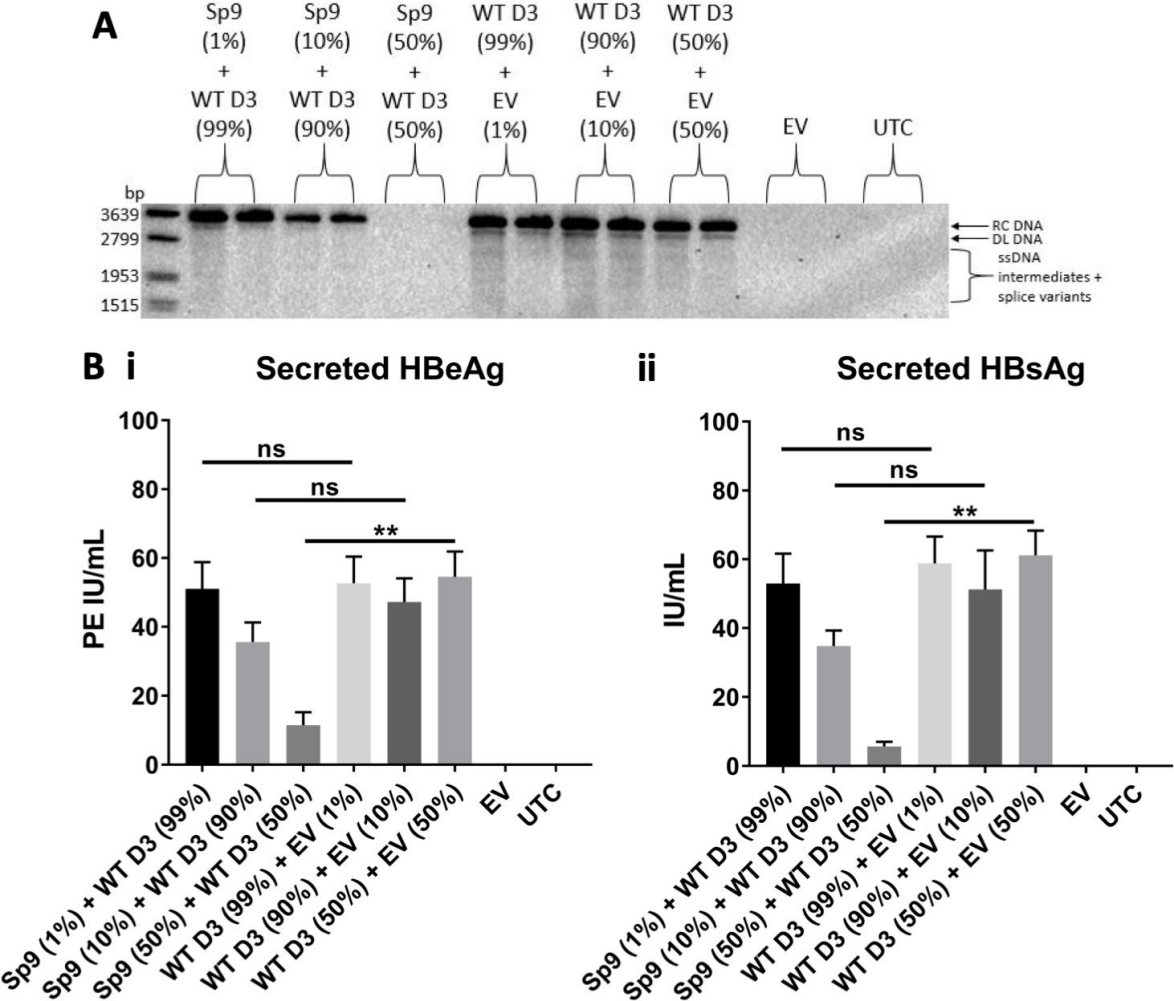
The effect of Sp9 on wild-type HBV replication was dose dependent. (**A)** Southern blot of intracellular core-associated DNA 5 days post-transfection of plasmids expressing Sp9 with WT HBV at different ratios in HepG2 cells. RC DNA, relaxed circular DNA; DL DNA, double-stranded linear DNA; ssDNA, single-stranded DNA. (**B**) Quantitative serology of secreted (i) HBeAg and (ii) HBsAg expression 5 days post-transfection of plasmids expressing Sp9 with WT HBV at different ratios in HepG2 cells. Error bars show SEM and *P*-values are produced from *t*-tests from three individual experiments. *, *P* < 0.05; **, *P* < 0.01; ***, *P* < 0.001; ns, not significant; PE, Paul-Ehrlich units; UTC, untransfected control; EV, empty vector.

To determine the impact of diluting Sp9 in the transfection mix on inhibition of wild-type HBV replication, Sp9 plasmid was transfected at different ratios, representing 1% and 10% of the transfection mix. A dose-dependent effect was observed, with as little as 10% of Sp9 impacting the level of wild-type intracellular core-associated DNA (Fig. 6A). A trend toward a dose-dependent effect was also observed for the level of secreted HBeAg and HBsAg, although this did not reach statistical significance (Fig. 6B).

### Sp9 expression reduced HBV replication in HepAD38 cells

**Fig 7:**
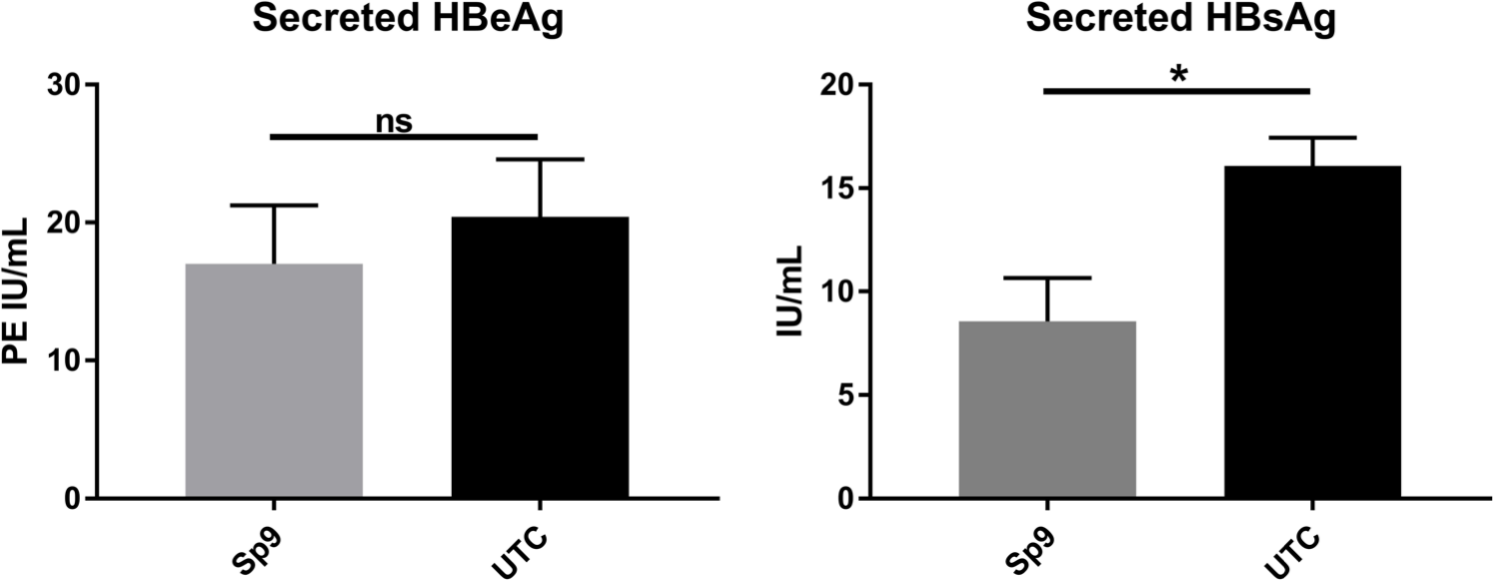
Sp9 expression reduced HBV replication in HepAD38 cells. Secreted HBeAg and HBsAg from HepAD38 cells 5 days post-transfection with plasmid expressing Sp9. Error bars show SEM and *P*-values are produced from *t*-tests from three individual experiments. *, *P* < 0.05; ns, not significant; UTC, untransfected control; PE, Paul-Ehrlich units.

To investigate Sp9’s impact on HBV replication in an HBV stable cell line, HepAD38 cells were transfected with plasmid expressing Sp9. Sp9 significantly reduced secreted HBsAg by 47% 5 days post-transfection (Fig. 7). A small decrease was also observed for secreted HBeAg, although this did not reach statistical significance (Fig. 7).

### Sp9 PC-S and core-S fusion protein contributed to Sp9’s impact on wild-type HBV replication

#### Co-transfection of Sp9 novel fusion protein expression plasmids with WT HBV

To investigate whether Sp9’s predicted novel fusion proteins (Fig. 1B) were involved in Sp9 mediated suppression of WT HBV replication, mammalian expression plasmids expressing the Sp9 novel fusion proteins were co-transfected with WT HBV D3 plasmid.

**Fig 8:**
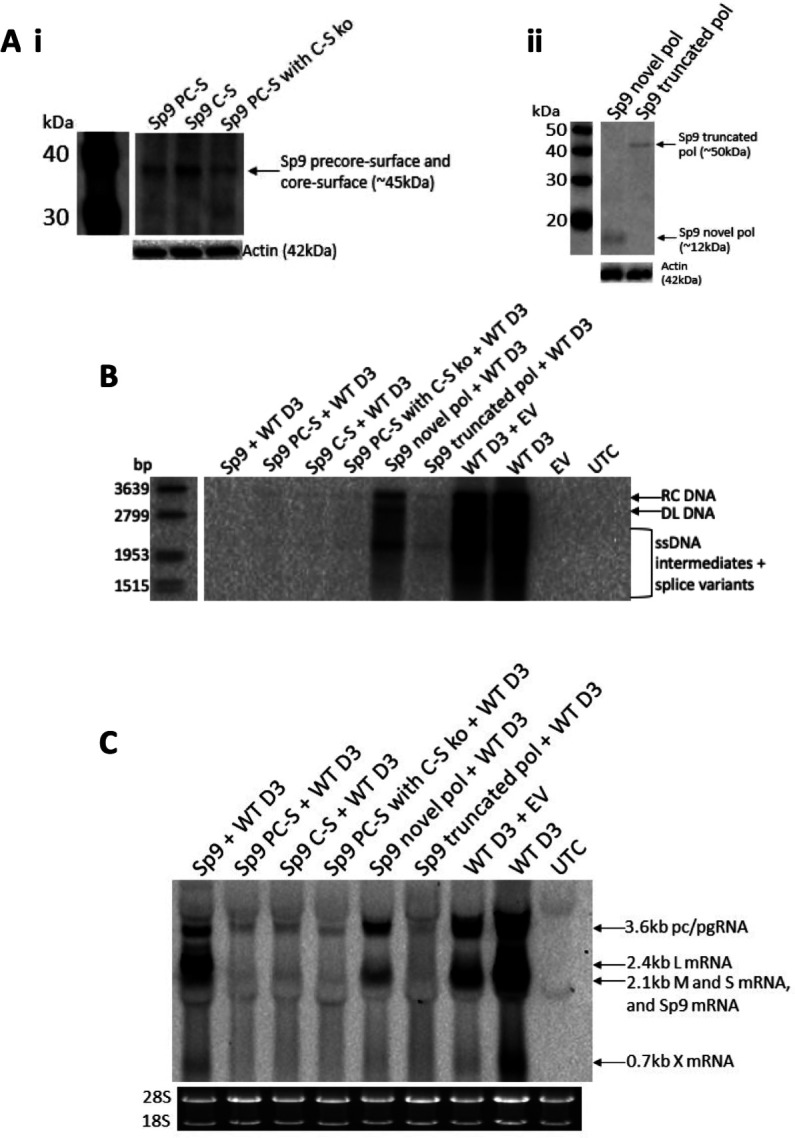
Expression plasmids encoding Sp9 precore-surface and core-surface fusion proteins reduced intracellular core-associated DNA and total RNA to a similar extent as Sp9. (**A)** Western blot of Sp9 novel (i) precore-surface, core-surface, precore-surface with core-surface knocked out, (ii) pol and truncated pol fusion proteins, probed with anti-flag antibody, 2 days post-transfection with Sp9 novel fusion protein expression plasmids. PC-S, precore-surface; C-S, core-surface (**B)** Southern blot of intracellular core-associated DNA 5 days post-transfection of plasmids expressing Sp9 novel fusion proteins with WT HBV D3. RC DNA, relaxed circular DNA; DL DNA, double-stranded linear DNA; ssDNA, single-stranded DNA. (**C)** Northern blot of intracellular RNA 5 days post-transfection of plasmids expressing Sp9 novel fusion proteins with WT HBV D3. pc/pgRNA, precore/pregenomic RNA; L, large surface; M, middle surface; S, small surface; HBx, X mRNA; UTC, untransfected control; EV, empty vector.

Western blotting confirmed that the Sp9 flag-tagged expression plasmids produced the expected novel fusion proteins (Fig. 8A). Co-transfection of WT HBV plasmid with expression plasmids encoding Sp9 PC-S, core-S, or a PC-S protein that had the core-S open reading frame knocked out completely abolished intracellular core-associated WT HBV DNA, to the same extent as Sp9 DNA co-transfected with WT HBV D3 plasmid (Fig. 8B). Expression of the Sp9 truncated pol protein reduced intracellular core-associated DNA to nearly the same extent as Sp9 itself, whereas expression of the Sp9 novel pol protein did not reduce intracellular core-associated DNA to the same extent as the complete Sp9 genome.

Analysis of intracellular RNA expression showed that co-transfection of expression plasmids encoding Sp9 PC-S, core-S, and PC-S protein that had the core-S open reading frame knocked out or truncated pol with WT HBV plasmid reduced the intracellular RNAs to a greater extent than Sp9 DNA transfected with WT HBV plasmid (Fig. 8C). In contrast to the findings for HBV DNA, co-transfection of expression plasmid expressing the Sp9 novel pol protein reduced the intracellular RNAs to a similar extent as Sp9 co-transfected with WT HBV plasmid.

#### Co-transfection of mutated Sp9 genome to prevent expression of Sp9 novel fusion proteins with WT HBV

**Fig 9:**
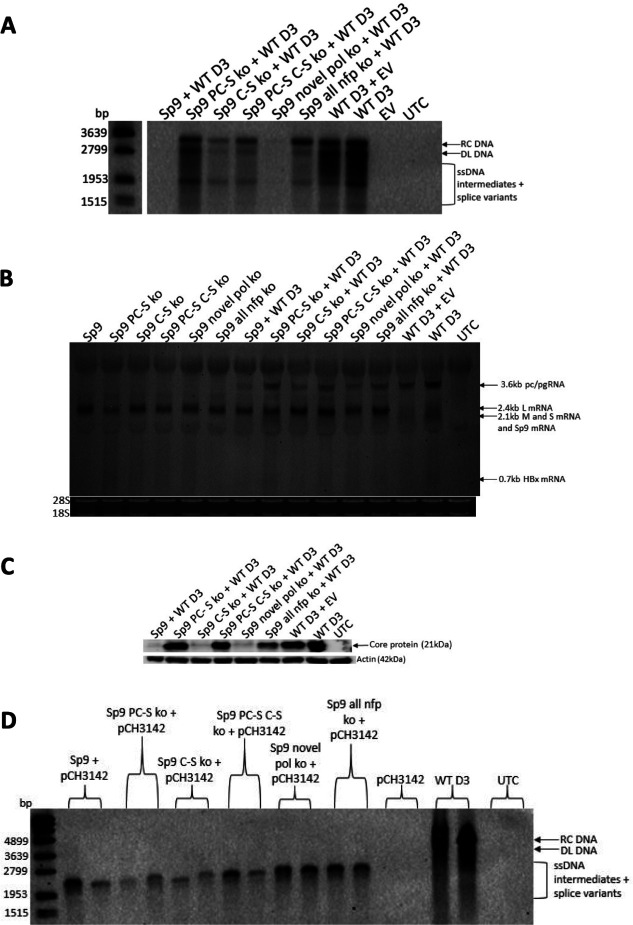
Knocking out Sp9 novel fusion proteins restored wild-type HBV replication. (**A)** Southern blot of intracellular core-associated DNA 5 days post-transfection of plasmids expressing mutant Sp9 clones with WT HBV D3. RC DNA, relaxed circular DNA; DL DNA, double-stranded linear DNA; ssDNA, single-stranded DNA. (**B)** Northern blot of intracellular RNA 5 days post-transfection of plasmids expressing mutant Sp9 clones with WT HBV D3. pc/pgRNA, precore/pregenomic RNA; L, large surface; M, middle surface; S, small surface; HBx, X mRNA. (**C)** Western blot of intracellular core, probed with in-house monoclonal antibody 1D8, expression 5 days post-transfection of plasmids expressing mutant Sp9 clones with WT HBV D3. (**D)** Southern blot of intracellular core-associated DNA 5 days post-transfection of plasmids expressing mutant Sp9 clones with pCH3142. PC-S, precore-surface; C-S, core-surface; ko, knockout; nfp, novel fusion protein; UTC, untransfected control; EV, empty vector.

Having shown that over-expression of Sp9 fusion proteins impacted HBV replication, it was next investigated whether knocking out expression of the fusion proteins in the “genome-length” Sp9 construct reduced the ability of Sp9 to impair WT HBV replication. Knocking out Sp9 PC-S novel fusion protein almost completely restored intracellular core-associated HBV DNA, whereas knocking out Sp9 core-S novel fusion protein or all the Sp9 fusion proteins only partially restored intracellular core-associated HBV DNA expression (Fig. 9A). However, knocking out the Sp9 novel pol protein did not restore intracellular core-associated HBV DNA expression. A similar effect was observed for intracellular RNA (Fig. 9B). Knocking out Sp9 PC-S novel fusion protein completely restored intracellular core expression, whereas knocking out Sp9 core-S novel fusion protein did not restore intracellular core expression (Fig. 9C). Co-transfection of the Sp9 mutant clones with pCH3142 confirmed they could replicate when the pol and core protein were provided *in trans* (Fig. 9D).

## DISCUSSION

This is the first study to investigate the replication phenotype of HBV splice variants Sp3 and Sp9, previously reported as the second most common HBV splice variants (3, 10, 25–28), and determine their impact on replication of wild-type HBV.

We showed that unlike Sp1, which only required the pol protein to be provided *in trans* to replicate, Sp3 and Sp9 also required *in trans* provision of the HBV core protein. This demonstrates that Sp3 novel core-pol fusion protein and Sp9 novel core-S fusion protein cannot replace the wild-type core protein to package spliced RNA. Furthermore, we showed that unlike other splice variants that have been studied to date, Sp3 has no effect on wild-type HBV replication. Of all splice variants identified thus far, generation of Sp3 RNA produces the largest intron (between nt 2067 and 489). Since Sp3 has no impact on wild-type HBV replication, this suggests that sequences within the large Sp3 intron that are present in other splice variants likely contribute to their inhibitory effect on wild-type HBV replication.

Previous studies have so far suggested two different mechanisms by which splice variants impact HBV replication—through the production of novel fusion proteins or by acting as non-coding RNA. For example, the Sp1 novel PC protein impacted wild-type HBV replication by preventing HBV nucleocapsid formation (15). Sp3 is predicted to produce a novel PC-pol and core-pol fusion protein (Fig. 1B), yet had no impact on wild-type HBV replication. This may be due to the Sp3 PC/core sequence being shorter than the Sp1 PC sequence, and so the protein may be missing regions essential to impact wild-type HBV replication. Sp10 suppression of replication by acting as non-coding RNA and interacting with the TATA-binding protein has been shown to be dependent on the region between nucleotides 432–832, encompassing part of the S and pol ORF (18). This region is absent from Sp3, supporting the notion that the regions essential for impacting wild-type HBV replication are removed during production of Sp3 RNA.

In contrast, Sp9 strongly decreased wild-type HBV replication in a co-transfection model, to a greater extent than previously reported for Sp1 (15, 22) and in an HBV stable cell line. This effect was largely dependent on the production of Sp9 PC-S fusion protein, as knocking out expression of this protein almost completely restored HBV DNA and completely restored HBV RNA and core expression. It is unclear if the Sp9 PC-S fusion protein impacts wild-type HBV nucleocapsid formation as has been reported for the Sp1 novel PC protein (15), or whether this protein acts similar to some isoforms of the wild-type precore protein that incorporate into nucleocapsids to impact intracellular core expression, RNA encapsidation, and DNA synthesis (40). Sp9 core-S fusion protein was also involved in Sp9 suppression of wild-type HBV replication, as knocking out this protein partially restored HBV DNA and RNA expression, although there was no restoration of HBV core expression, suggesting that the Sp9 core-S fusion protein may interfere with wild-type HBV replication through different mechanisms to the Sp9 PC-S fusion protein. Sp9 also encodes the sequence (nt 432 to 832) essential for Sp10 to interfere with HBV RNA transcription (18). It remains to be determined whether Sp9 RNA could similarly act as a non-coding RNA that interferes with HBV RNA transcription or whether the novel fusion proteins interfere with HBV RNA transcription.

Our findings support previous studies that showed splice variants impact wild-type HBV replication, despite differences in our experimental design to better reflect the potential natural effect of splice variants on wild-type HBV replication (15, 18). For example, we showed the impact of splice variants on wild-type HBV replication using RNA transcribed with HBV promoters, rather than CMV-driven expression plasmids (15). In addition, we confirmed that genotype A2-derived Sp10 reduced HBV replication after co-transfection with a genotype A2 WT HBV plasmid, confirming previous studies which co-transfected Sp10 with a genotype D WT HBV plasmid (18).

One limitation of our study is that transfection of plasmid was used to express Sp9 in hepatoma cells which may not accurately reflect native expression of spliced RNAs and their putative novel proteins. As HBV splice variants require HBV proteins *in trans* to replicate, current cell culture models do not permit study of the impact of HBV splice variants on HBV replication in an infection setting. A further limitation is that our study was exclusively cell culture-based; it remains to be determined whether a similar effect on wild-type HBV replication would be mediated by splice variants and novel fusion proteins in murine models or in people living with chronic HBV. Sp9 RNA has been identified at low levels in chronic hepatitis B (CHB) patient tissue and tumor samples at very small amounts (<10% of total HBV RNA), which differs between genotypes(10). We have shown *in vitro* that levels of up to 10% of Sp9 decreased wild-type HBV replication; however, whether this reflects the impact of Sp9 in the setting of CHB remains unknown.

Our study provides further evidence that HBV splice variants impact replication of wild-type HBV. Although Sp3 had no effect on wild-type HBV replication, Sp9 had a profound impact when present at high levels in cell culture, which was largely due to expression of novel fusion proteins. Although this effect was less pronounced when the proportion of Sp9 to wild-type HBV more closely reflected the levels observed in CHB patients, the impact of Sp9 fusion protein expression in CHB patients where HBV may have been present for decades is unknown. This should be further explored using longitudinal studies of patient serum and in *in vivo* studies. Although the biological significance of Sp3 and Sp9 is largely unknown, frequent detection of Sp9 in clinical samples (10, 29), together with our findings that Sp9 strongly reduces wild-type HBV replication *in vitro,* suggests biological importance to HBV infection that justifies further examination. Taken together, the role of splice variants in HBV replication remains largely unclear; however, our findings show that the novel fusion proteins produced by splice variants impact HBV replication and warrant further investigation.

## Data Availability

All data are available in the main text and supplementary materials.

## SUPPLEMENTAL MATERIAL

Fig. S1 to S3 jvi.01538-23-s0001.docx Map of HBV clones used in study, viability of transfected cells, and sequences of Sp3 and Sp9 clones.

ASM does not own the copyrights to Supplemental Material that may be linked to, or accessed through, an article. The authors have granted ASM a non-exclusive, world-wide license to publish the Supplemental Material files. Please contact the corresponding author directly for reuse.
