# Supplementary material for: The in vitro replication phenotype of hepatitis B virus (HBV) splice variants Sp3 and Sp9 and their impact on wild-type HBV replication
Source: J Virol. 2024 Mar 19;98(4):e01538-23. doi: 10.1128/jvi.01538-23 (PMC11019940; doi:10.1128/jvi.01538-23)
Supplement: Fig. S1 to S3 — Map of HBV clones used in study, viability of transfected cells, and sequences of Sp3 and Sp9 clones. [file jvi.01538-23-s0001.docx]

**Supplementary material:**

**Supplementary Figure 1:**


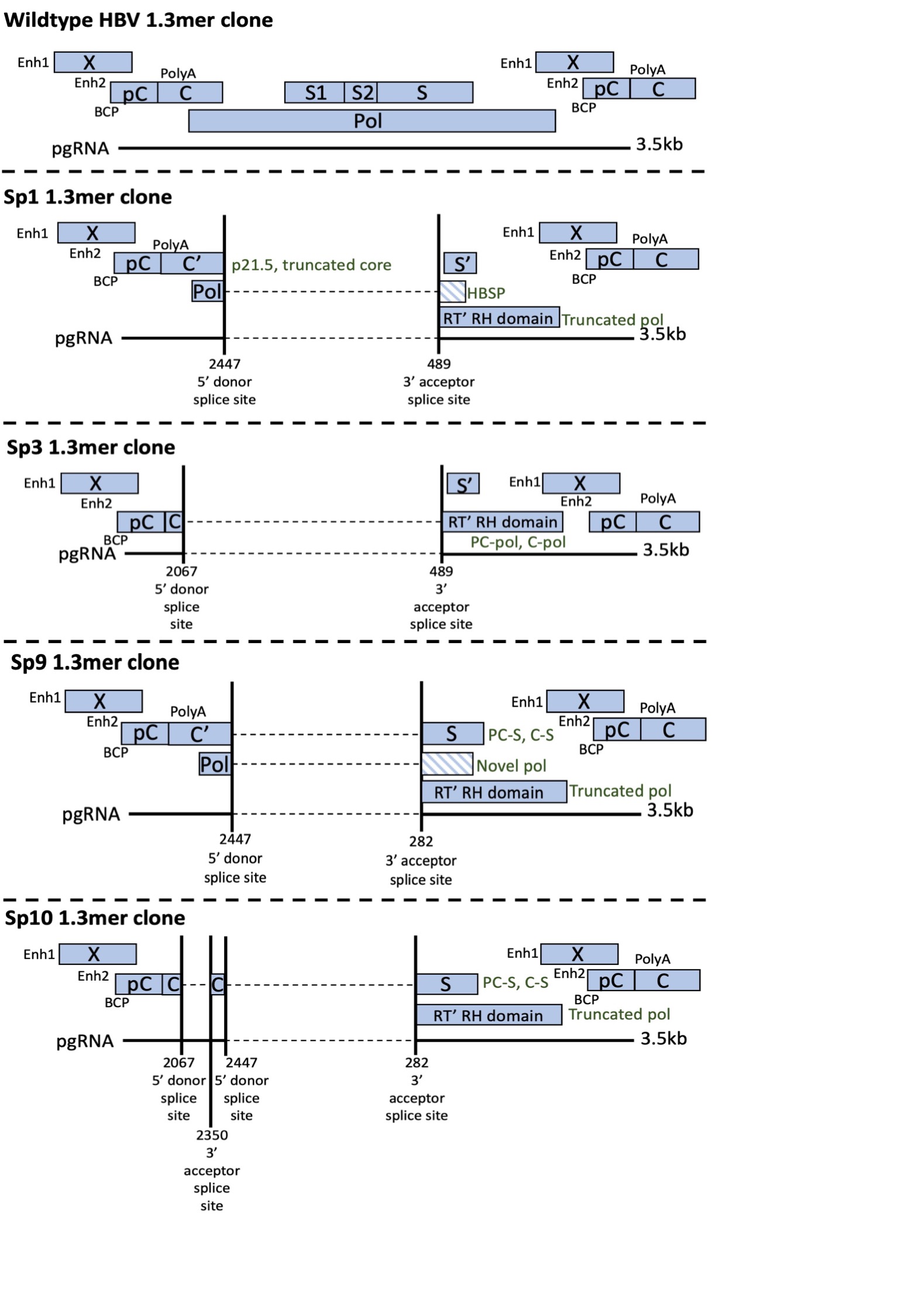


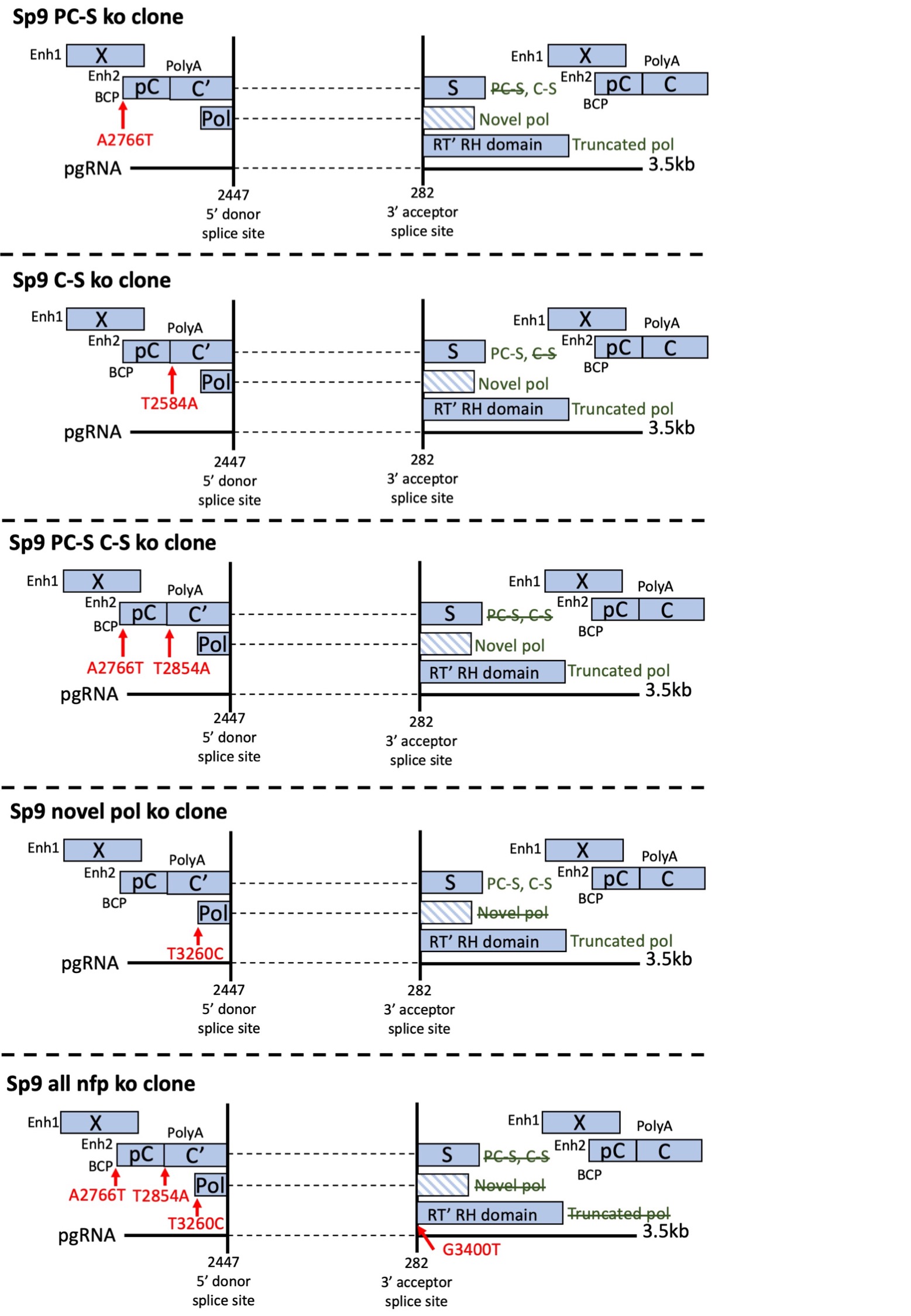


**Figure S1. Map of HBV 1.3mer clones used in this study.** Blue squares indicate open reading frames. Shaded blue squares indicate new open reading frame. Novel fusion proteins derived from splicing are indicated in green. Novel fusion proteins that are not produced due to mutation of the start codon are indicated with a crossed line. Mutations of the start codons are indicated in red. pC: precore, C: core, pol: polymerase, RT’ RH domain: truncated reverse transcriptase RNaseH domain, pgRNA: pregenomic RNA, X: HBx, S: small surface, S1: preS1, S2: preS2, polyA: polyA tail, Enh1: Enhancer 1, Enh2: Enhancer 2, BCP: basal core promoter, ko: knockout, PC-S: precore-surface, C-S: core-surface, nfp: novel fusion protein, ‘: truncated open reading frame.

**Supplementary Figure 2:**


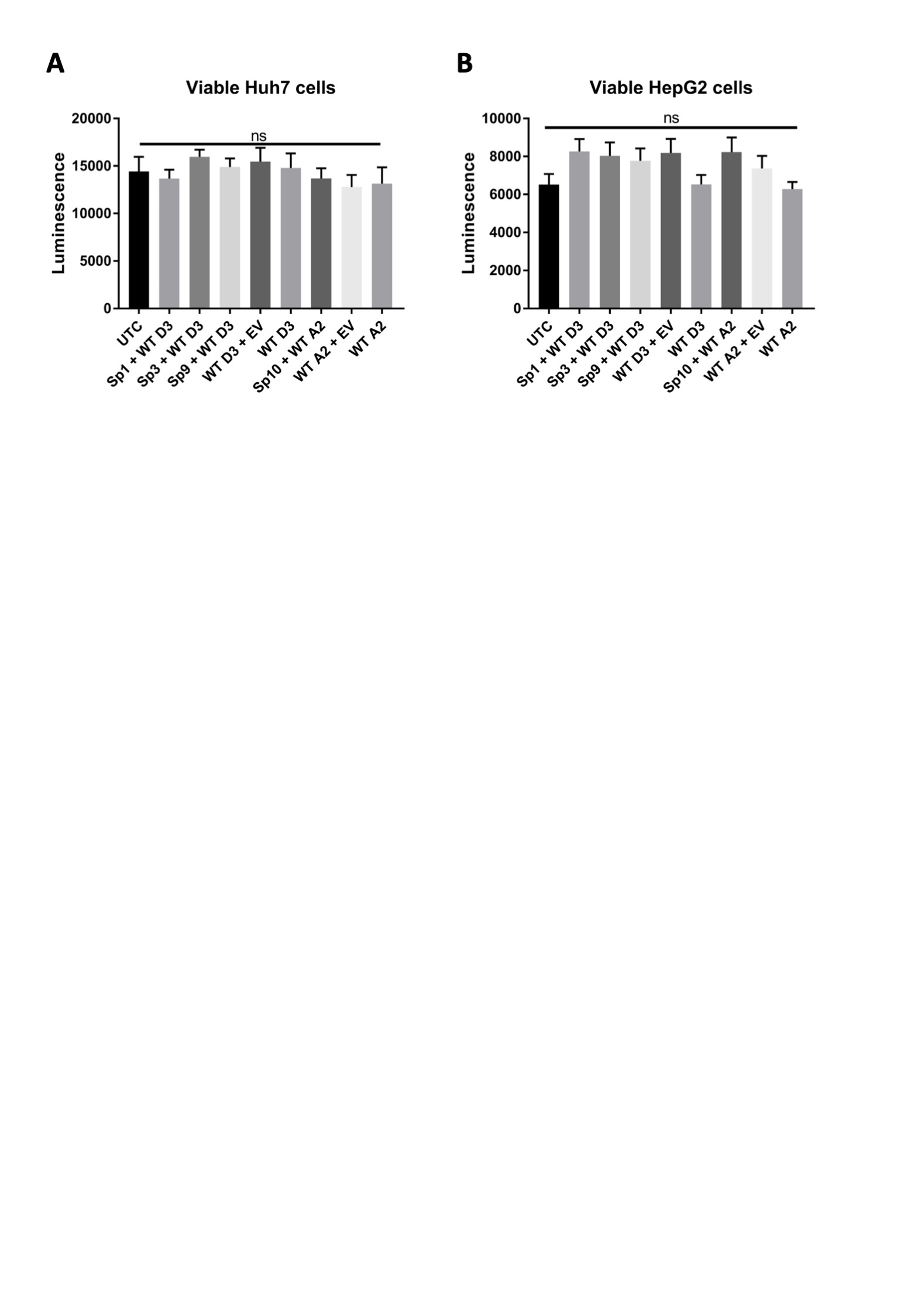
 **Figure S2.** **Viability of cells transfected with HBV splice variants.**

Cell viability of **(A)** Huh7 and **(B)** HepG2 cells expressing Sp1, Sp3, Sp9 and Sp10 with WT HBV compared to an untransfected control (UTC). Error bars show SEM and p-values are produced from t-tests from three individual experiments. ns: not significant. EV: empty vector.

**Supplementary Figure 3:**

**Sp3 1.3mer**

GGGCCCTGCAGGTCGACAAGCTTGTATTCAATCTAAGCAGGCTTTCACTTTCTCGCCAACTTACAAGGCCTTTCTGTGTAAACAATACCTGAACCTTTACCCCGTTGCCCGGCAACGGCCAGGTCTGTGCCAAGTGTTTGCTGACGCAACCCCCACTGGCTGGGGCTTGGTCATGGGCCATCAGCGCATGCGTGGAACCTTTTCGGCTCCTCTGCCGATCCATACTGCGGAACTCCTAGCCGCTTGTTTTGCTCGCAGCAGGTCTGGAGCAAACATTATCGGGACTGATAACTCTGTTGTCCTATCCCGCAAATATACATCGTTTCCATGGCTGCTAGGCTGTGCTGCCAACTGGATCCTGCGCGGGACGTCCTTTGTTTACGTCCCGTCGGCGCTGAATCCTGCGGACGACCCTTCTCGGGGTCGCTTGGGACTCTCTCGTCCCCTTCTCCGTCTGCCGTTCCGACCGACCACGGGGCGCACCTCTCTTTACGCGGACTCCCCGTCTGTGCCTTCTCATCTGCCGGACCGTGTGCACTTCGCTTCACCTCTGCACGTCGCATGGAGACCACCGTGAACGCCCACCAAATATTGCCCAAGGTCTTACATAAGAGGACTCTTGGACTCTCAGCAATGTCAACGACCGACCTTGAGGCATACTTCAAAGACTGTTTGTTTAAAGACTGGGAGGAGTTGGGGGAGGAGATTAGGTTAAAGGTCTTTGTACTAGGAGGCTGTAGGCATAAATTGGTCTGCGCACCAGCACCATGCAACTTTTTCACCTCTGCCTAATCATCTCTTGTTCATGTCCTACTGTTCAAGCCTCCAAGCTGTGCCTTGGGTGGCTTTGGGGCATGGACATCGACCCTTATAAAGAATTTGGAGCTACTGTGGAGTTACTCTCGTTTTTGCCTTCTGACTTCTTTCCTTCAGTACGAGATCTTCTAGATACCGCCTCAGCTCTGTATCGGGAAGCCTTAGAGTCTCCTGAGCATTGTTCACCTCACCATACTGCACTCAGGATCCTCAACAACCAGCACGGGACCATGCCGGACCTGCAAGACTACTGCTCAAGGAACCTCTATGTATCCCTCCTGTTGCTGTACCAAACCTTCGGACGGAAATTGCACCTGTATTCCCATCCCATCATCCTGGGCTTTCGGAAAATTCCTATGGGAGTGGGCCTCAGCCCGTTTCTCCTGGCTCAGTTTACTAGTGCCATTTGTTCAGTGGTTCGTAGGGCTTTCCCCCACTGTTTGGCTTTCAGTTATATGGATGATGTGGTATTGGGGGCCAAGTCTGTACAGCATCTTGAGTCCCTTTTTACCGCTGTTACCAATTTTCTTTTGTCTTTGGGTATACATTTAAACCCTAACAAAACAAAGAGATGGGGTTACTCTCTAAATTTTATGGGTTATGTCATTGGATGTTATGGGTCCTTGCCACAAGAACACATCATACAAAAAATCAAAGAATGTTTTAGAAAACTTCCTATTAACAGGCCTATTGATTGGAAAGTATGTCAACGAATTGTGGGTCTTTTGGGTTTTGCTGCCCCTTTTACACAATGTGGTTATCCTGCGTTGATGCCTTTGTATGCATGTATTCAATCTAAGCAGGCTTTCACTTTCTCGCCAACTTACAAGGCCTTTCTGTGTAAACAATACCTGAACCTTTACCCCGTTGCCCGGCAACGGCCAGGTCTGTGCCAAGTGTTTGCTGACGCAACCCCCACTGGCTGGGGCTTGGTCATGGGCCATCAGCGCATGCGTGGAACCTTTTCGGCTCCTCTGCCGATCCATACTGCGGAACTCCTAGCCGCTTGTTTTGCTCGCAGCAGGTCTGGAGCAAACATTATCGGGACTGATAACTCTGTTGTCCTATCCCGCAAATATACATCGTTTCCATGGCTGCTAGGCTGTGCTGCCAACTGGATCCTGCGCGGGACGTCCTTTGTTTACGTCCCGTCGGCGCTGAATCCTGCGGACGACCCTTCTCGGGGTCGCTTGGGACTCTCTCGTCCCCTTCTCCGTCTGCCGTTCCGACCGACCACGGGGCGCACCTCTCTTTACGCGGACTCCCCGTCTGTGCCTTCTCATCTGCCGGACCGTGTGCACTTCGCTTCACCTCTGCACGTCGCATGGAGACCACCGTGAACGCCCACCAAATATTGCCCAAGGTCTTACATAAGAGGACTCTTGGACTCTCAGCAATGTCAACGACCGACCTTGAGGCATACTTCAAAGACTGTTTGTTTAAAGACTGGGAGGAGTTGGGGGAGGAGATTAGGTTAAAGGTCTTTGTACTAGGAGGCTGTAGGCATAAATTGGTCTGCGCACCAGCACCATGCAACTTTTTCACCTCTGCCTAATCATCTCTTGTTCATGTCCTACTGTTCAAGCCTCCAAGCTGTGCCTTGGGTGGCTTTGGGGCATGGACATCGACCCTTATAAAGAATTTGGAGCTACTGTGGAGTTACTCTCGTTTTTGCCTTCTGACTTCTTTCCTTCAGTACGAGATGCTAGCCCGGG

**Sp9 1.3mer**

TGCAGGGCCCTGCAGGTCGACAAGCTTGTATTCAATCTAAGCAGGCTTTCACTTTCTCGCCAACTTACAAGGCCTTTCTGTGTAAACAATACCTGAACCTTTACCCCGTTGCCCGGCAACGGCCAGGTCTGTGCCAAGTGTTTGCTGACGCAACCCCCACTGGCTGGGGCTTGGTCATGGGCCATCAGCGCATGCGTGGAACCTTTTCGGCTCCTCTGCCGATCCATACTGCGGAACTCCTAGCCGCTTGTTTTGCTCGCAGCAGGTCTGGAGCAAACATTATCGGGACTGATAACTCTGTTGTCCTATCCCGCAAATATACATCGTTTCCATGGCTGCTAGGCTGTGCTGCCAACTGGATCCTGCGCGGGACGTCCTTTGTTTACGTCCCGTCGGCGCTGAATCCTGCGGACGACCCTTCTCGGGGTCGCTTGGGACTCTCTCGTCCCCTTCTCCGTCTGCCGTTCCGACCGACCACGGGGCGCACCTCTCTTTACGCGGACTCCCCGTCTGTGCCTTCTCATCTGCCGGACCGTGTGCACTTCGCTTCACCTCTGCACGTCGCATGGAGACCACCGTGAACGCCCACCAAATATTGCCCAAGGTCTTACATAAGAGGACTCTTGGACTCTCAGCAATGTCAACGACCGACCTTGAGGCATACTTCAAAGACTGTTTGTTTAAAGACTGGGAGGAGTTGGGGGAGGAGATTAGGTTAAAGGTCTTTGTACTAGGAGGCTGTAGGCATAAATTGGTCTGCGCACCAGCACCATGCAACTTTTTCACCTCTGCCTAATCATCTCTTGTTCATGTCCTACTGTTCAAGCCTCCAAGCTGTGCCTTGGGTGGCTTTGGGGCATGGACATCGACCCTTATAAAGAATTTGGAGCTACTGTGGAGTTACTCTCGTTTTTGCCTTCTGACTTCTTTCCTTCAGTACGAGATCTTCTAGATACCGCCTCAGCTCTGTATCGGGAAGCCTTAGAGTCTCCTGAGCATTGTTCACCTCACCATACTGCACTCAGGCAAGCAATTCTTTGCTGGGGGGAACTAATGACTCTAGCTACCTGGGTGGGTGTTAATTTGGAAGATCCAGCGTCTAGAGACCTAGTAGTCAGTTATGTCAACACTAATATGGGCCTAAAGTTCAGGCAACTCTTGTGGTTTCACATTTCTTGTCTCACTTTTGGAAGAGAAACAGTTATAGAGTATTTGGTGTCTTTCGGAGTGTGGATTCGCACTCCTCCAGCTTATAGACCACCAAATGCCCCTATCCTATCAACACTTCCGGAGACTACTGTTGTTAGACGACGAGGCAGGTCCCCTAGAAGAAGAACTCCCTCGCCTCGCAGACGAAGGTCTCAATCGCCGCGTCGCAGAAGATCTCAATCTCGGGAATCTCAATGGGGAACTACCGTGTGTCTTGGCCAAAATTCGCAGTCCCCAACCTCCAATCACTCACCAACCTCTTGTCCTCCAACTTGTCCTGGTTATCGCTGGATGTGTCTGCGGCGTTTTATCATCTTCCTCTTCATCCTGCTGCTATGCCTCATCTTCTTGTTGGTTCTTCTGGACTATCAAGGTATGTTGCCCGTTTGTCCTCTAATTCCAGGATCCTCAACAACCAGCACGGGACCATGCCGGACCTGCACGACTACTGCTCAAGGAACCTCTATGTATCCCTCCTGTTGCTGTACCAAACCTTCGGACGGAAATTGCACCTGTATTCCCATCCCATCATCCTGGGCTTTCGGAAAATTCCTATGGGAGTGGGCCTCAGCCCGTTTCTCCTGGCTCAGTTTACTAGTGCCATTTGTTCAGTGGTTCGTAGGGCTTTCCCCCACTGTTTGGCTTTCAGTTATATGGATGATGTGGTATTGGGGGCCAAGTCTGTACAGCATCTTGAGTCCCTTTTTACCGCTGTTACCAATTTTCTTTTGTCTTTGGGTATACATTTAAACCCTAACAAAACAAAGAGATGGGGTTACTCTCTAAATTTTATGGGTTATGTCATTGGATGTTATGGGTCCTTGCCACAAGAACACATCATACAAAAAATCAAAGAATGTTTTAGAAAACTTCCTATTAACAGGCCTATTGATTGGAAAGTATGTCAACGAATTGTGGGTCTTTTGGGTTTTGCTGCCCCTTTTACACAATGTGGTTATCCTGCGTTGATGCCTTTGTATGCATGTATTCAATCTAAGCAGGCTTTCACTTTCTCGCCAACTTACAAGGCCTTTCTGTGTAAACAATACCTGAACCTTTACCCCGTTGCCCGGCAACGGCCAGGTCTGTGCCAAGTGTTTGCTGACGCAACCCCCACTGGCTGGGGCTTGGTCATGGGCCATCAGCGCATGCGTGGAACCTTTTCGGCTCCTCTGCCGATCCATACTGCGGAACTCCTAGCCGCTTGTTTTGCTCGCAGCAGGTCTGGAGCAAACATTATCGGGACTGATAACTCTGTTGTCCTATCCCGCAAATATACATCGTTTCCATGGCTGCTAGGCTGTGCTGCCAACTGGATCCTGCGCGGGACGTCCTTTGTTTACGTCCCGTCGGCGCTGAATCCTGCGGACGACCCTTCTCGGGGTCGCTTGGGACTCTCTCGTCCCCTTCTCCGTCTGCCGTTCCGACCGACCACGGGGCGCACCTCTCTTTACGCGGACTCCCCGTCTGTGCCTTCTCATCTGCCGGACCGTGTGCACTTCGCTTCACCTCTGCACGTCGCATGGAGACCACCGTGAACGCCCACCAAATATTGCCCAAGGTCTTACATAAGAGGACTCTTGGACTCTCAGCAATGTCAACGACCGACCTTGAGGCATACTTCAAAGACTGTTTGTTTAAAGACTGGGAGGAGTTGGGGGAGGAGATTAGGTTAAAGGTCTTTGTACTAGGAGGCTGTAGGCATAAATTGGTCTGCGCACCAGCACCATGCAACTTTTTCACCTCTGCCTAATCATCTCTTGTTCATGTCCTACTGTTCAAGCCTCCAAGCTGTGCCTTGGGTGGCTTTGGGGCATGGACATCGACCCTTATAAAGAATTTGGAGCTACTGTGGAGTTACTCTCGTTTTTGCCTTCTGACTTCTTTCCTTCAGTACGAGATGCTAGCCCGGGCGAT
